# Global trend of *Plasmodium malariae* and *Plasmodium ovale* spp. malaria infections in the last two decades (2000–2020): a systematic review and meta-analysis

**DOI:** 10.1186/s13071-021-04797-0

**Published:** 2021-06-03

**Authors:** Joseph Hawadak, Rodrigue Roman Dongang Nana, Vineeta Singh

**Affiliations:** 1 ICMR-National Institute of Malaria Research, Dwarka, Sector 8, New-Delhi, 110077 India; 2 Institute of Medical Research and Medicinal Plants Studies, PO Box 13033, Yaoundé, Cameroon

**Keywords:** Malaria infections, *Plasmodium malariae*, *Plasmodium ovale* spp., Prevalence, Meta-analysis

## Abstract

**Background:**

Recent studies indicate that the prevalence of non-falciparum malaria, including* Plasmodium malariae* and *Plasmodium ovale* spp., is increasing, with some complications in infected individuals. The aim of this review is to provide a better understanding of the malaria prevalence and disease burden due to *P. malariae* and *P. ovale* spp.

**Methods:**

The Preferred Reporting Items for Systematic Reviews and Meta-Analyses (PRISMA) guidelines and the Joanna Briggs Institute prevalence study assessment tool were used to select and evaluate the studies, respectively. Six databases: PubMed, WHOLIS, Wiley Library, ScienceDirect, Web of Science and Google Scholar were used to screen articles published during the period January 2000–December 2020. The pooled prevalence estimates for *P. malariae* and *P. ovale* spp. were analysed using a random-effects model and the possible sources of heterogeneity were evaluated through subgroup analysis and meta-regression.

**Results:**

Out of the 3297 studies screened, only 113 studies were included; among which 51.33% were from the African Region. The *P. malariae* and *P. ovale* spp. pooled prevalence were 2.01% (95% CI 1.31–2.85%) and 0.77% (95% CI 0.50–1.10%) respectively, with the highest prevalence in the African Region. *P. malariae* was equally distributed among adults (2.13%), children (2.90%) and pregnant women (2.77%) (*p* = 0.862), whereas *P. ovale* spp. was more prevalent in pregnant women (2.90%) than in children ≤ 15 years (0.97%) and in patients > 15 years old (0.39%) (*p* = 0.021). In this review, data analysis revealed that *P. malariae* and *P. ovale* spp. have decreased in the last 20 years, but not significantly, and these species were more commonly present with other *Plasmodium* species as co-infections. No difference in prevalence between symptomatic and asymptomatic patients was observed for either *P. malariae* or *P. ovale* spp.

**Conclusion:**

Our analysis suggests that knowledge of the worldwide burden of *P. malariae* and *P. ovale* spp. is very important for malaria elimination programmes and a particular focus towards improved tools for monitoring transmission for these non-falciparum species should be stressed upon to deal with increased infections in the future.

**Graphic Abstract:**

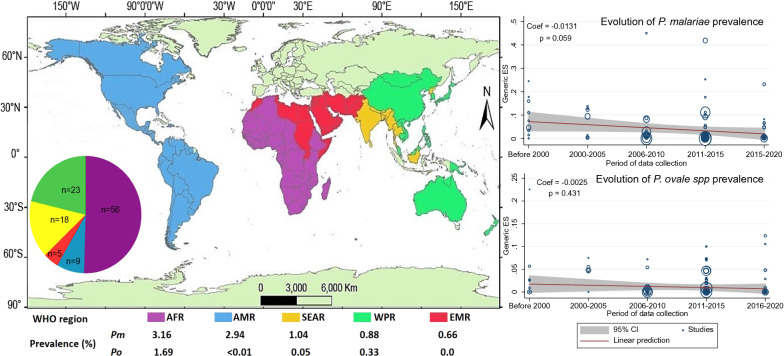

**Supplementary Information:**

The online version contains supplementary material available at 10.1186/s13071-021-04797-0.

## Background

Malaria is an infectious disease caused by parasites of the genus *Plasmodium* and transmitted by infected *Anopheles* female mosquitoes in tropical and subtropical countries. This disease is a major public health problem globally, with 229 million estimated cases in 2019, mostly in African and South-East Asia regions [1]. Several international programmes have been launched to reduce and/or eradicate malaria worldwide, and considerable progress has been made over the past two decades, resulting in a decrease in the global incidence of malaria from 363 to 225 cases per 1000 population during the years 2000 to 2019 [1]. Unfortunately, recent reports of the emergence of artemisinin resistance in South-East Asia in the Greater Mekong Subregion adds another challenge hindering the efforts of malaria control programmes [2–4].

Among all human *Plasmodium* spp., *P. falciparum* and *P. vivax* receive particular scientific research interest, as they are responsible for the majority of malaria infections and disease severity in human patients, whereas *P. ovale* spp. and *P. malariae* remain neglected tropical diseases due to their low prevalence and still lower disease severity as compared to falciparum malaria [3, 5, 6]. The clinical features due to *P. malariae* and *P. ovale* spp. are thus poorly known, as very few reported studies are available compared to other human *Plasmodium* spp. [7]. *P. ovale* spp. are recognized to generally induce weak parasitaemia in the infected host, are very often responsible for relapses and sometimes have also been identified in certain renal complications [8]. In recent years, a steady rise in malaria infection cases associated with *P. ovale* spp. and *P. malariae* has been observed in many parts of sub-Saharan Africa and South America, with their prevalence being around 10% and 20%, respectively, in both symptomatic and asymptomatic malaria infections, causing concern for elimination programmes [9–14]. This increase in prevalence underscores the need for attention towards them, and therefore knowledge about their demographic distribution and associated morbidity is essential for understanding their epidemiology. In this review article, we have focused on estimating the disease burden and the epidemiological aspects of malaria infections caused by *P. malariae* and *P. ovale* spp., highlighting the need for increased attention towards these species for successful malaria elimination programmes.

## Methods

### Search strategy

The Preferred Reporting Items for Systematic Reviews and Meta-Analyses (PRISMA) guidelines were used to select studies for this review [15], and six databases (PubMed, WHOLIS, Wiley Library, ScienceDirect, Web of Science and Google Scholar) were explored to access articles (Additional file 1). Although the search strategy differs slightly from one database to another, a systematic computerized strategy was adopted using MeSH terms and in combination with Boolean operators: [“malaria” OR “*Plasmodium*” AND (“malariae” OR “ovale” OR “curtisi” OR “wallikeri”) AND (“prevalence” OR “epidemiology” OR “proportion” OR “frequency”) AND (“PCR” OR “molecular”)] (Additional file 2). In addition, a manual Google search and filtering of the reference lists of included studies were performed to access additional articles. The search included articles in both English and French languages published between January 2000 and December 2020.

### Selection of studies and data extraction

All epidemiological studies that reported the prevalence of *P. malariae* and *P. ovale* spp. using molecular techniques as a species identification method were considered in this review. Only research articles published in peer-reviewed journals were included, and studies with no clear epidemiological data on *Plasmodium* spp. were excluded from the review. All articles were imported into Zotero bibliographic reference management software, and duplicate files were deleted. A data extraction form with the name of the first author, year of publication, period of data collection, geographical location (continent and country), target group (subject and subject status), type of study, sample size, sampling technique, diagnostic method, target gene and a positive result for each human *Plasmodium* species was prepared in a Microsoft Excel spreadsheet. The data files extracted independently by the first two authors were systematically evaluated for any inconsistencies, and any further discrepancies were resolved after discussion with the team supervisor.

### Evaluation of criteria for inclusion of studies

The quality and risk of bias in included studies were assessed using an adapted version of the Joanna Briggs Institute (JBI) prevalence study assessment tool [16]. The evaluation criteria were based on eight parameters: (i) appropriate sample frame, (ii) appropriate sampling technique, (iii) adequate sample size, (iv) description of the study subject and settings in detail, (v) sufficient data analysis, (vi) use of validated methods for the conditions identified, (vii) valid measurement of the condition for all participants and (viii) use of appropriate statistical analysis. For each evaluation item, a score of 1 was awarded if found in the article; zero was given if not, and no score if the evaluation tool was unclear or not applicable to the study. Studies that received a score ≥ 5 were considered acceptable for final inclusion in the study (Additional file 3: Table S1).

### Data analysis

Data analysis was performed with *Metaprop* command [17] in Stata 16.1 software (StataCorp LLC, USA). The combined prevalence and 95% confidence intervals (CIs) were computed using a random-effects model with Freeman-Tukey double arcsine transformation and restricted maximum likelihood method for stabilization and estimating the covariance. Heterogeneity between studies was assessed using *I*^2^, whose values of 25%, 50% and 75% were considered as low, moderate and high degrees of heterogeneity, respectively. The risk difference (RD) was performed to compare the occurrences of *P. malariae/P. ovale* spp. in mono-infections and co-infections with other species using OpenMeta[Analyst] software. Publication bias was also assessed using a funnel diagram by visually inspecting the plots and exact Egger’s test for small-study effects. Possible sources of heterogeneity were assessed using subgroup analysis and meta-regression. We computed a subgroup analysis including only studies conducted in defined groups: (i) pregnant women, (ii) children ≤ 15 years old and (iii) participants > 15 years old. In the pregnant women group, when several samples were taken (peripheral blood, umbilical cord and/or placenta), only the results of the peripheral blood were used in the meta-analysis. A meta-regression with the sample size, date of publication and period of data collection as a covariate was performed to evaluate whether they were confounders of the *P. malaria* and/or *P. ovale* spp. pooled prevalence. All statistical analyses were considered statistically significant for values of *p* < 0.05.

## Results

### Study selection and identification

**Fig. 1:**
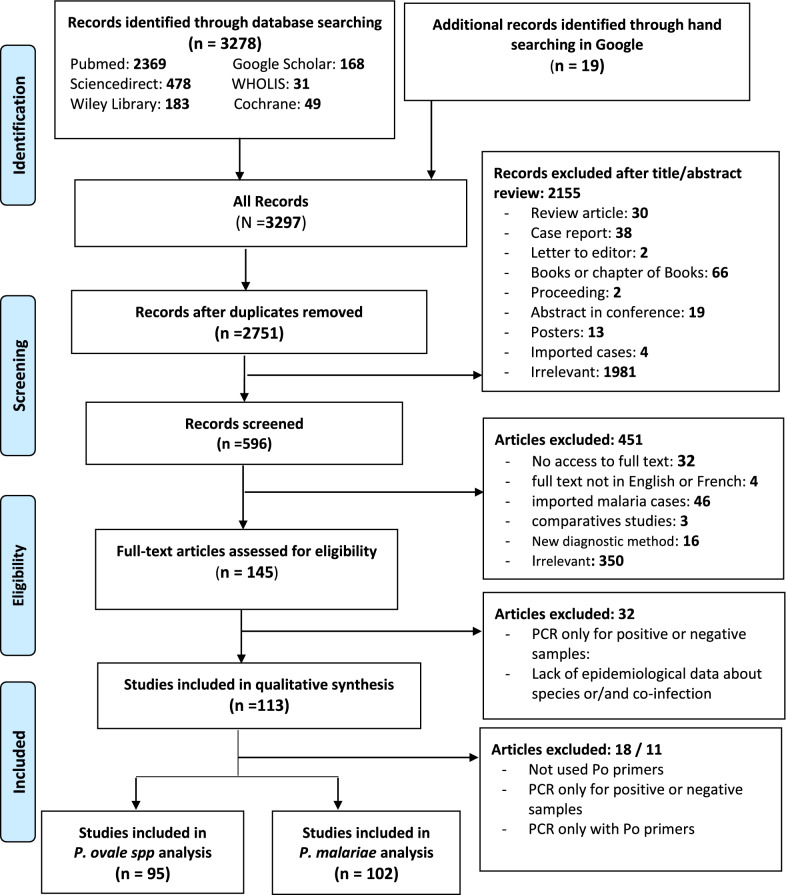
PRISMA flow chart of study selection for review

A total of 3297 citations were screened initially, where 3278 were obtained from the six selected databases and 19 others through the manual Google search for inclusion in this review study. Five hundred and forty-six were duplicates and hence deleted. From the 2751 remaining articles, 2155 were excluded after reviewing the abstract. and a further 451 studies were excluded for one of the following reasons: (i) no access to the full text (*n* = 32), (ii) article in a language other than English or French (*n* = 4), (iii) epidemiological data on imported malaria cases (*n* = 46), (iv) comparative studies or diagnosis made by a new method (*n* = 19) and (v) not related to our study framework (*n* = 350). Of the remaining 145 articles, 32 were excluded because of their methodology or a lack of epidemiological information (Additional file 3: Table S2). Finally, 113 studies involving 195,065 subjects were considered for the systematic review (Fig. 1). Ninety-five and 102 studies were included in quantitative analysis of *P. ovale* spp. and *P. malariae* prevalence, respectively. The characteristics of the studies included in this review are summarized in Additional file 3: Table S3.

### Characteristics of the included studies

**Fig. 2:**
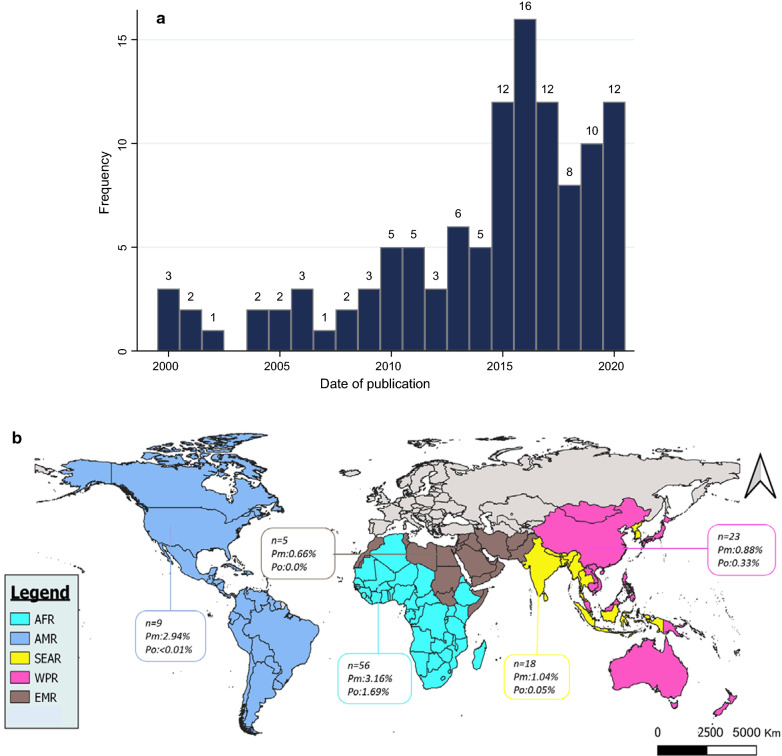
**a** Distribution of included studies depending on the year. **b** Map of WHO regions where included studies were conducted. The regionalization used on this map is that of the WHO Global Health Observatory map. WHO Member States are grouped into six regions. The European region is not represented here, as it was not included in our study. (AFR: African Region, WPR: Western Pacific Region AMR: Region of the Americas, SEAR: South-East Asia Region, EMR: Eastern Mediterranean Region). n: number of included studies in each region. *Pm: Plasmodium malariae, Po: Plasmodium ovale* spp.

Of the 113 studies included in our review, more than half (51.33%; *n* = 58) were published within the last 5 years, but no study was published in the year 2003 (Fig. 2a). The epidemiological studies included in this review covered five out of six pre-classified World Health Organization (WHO) health regions, with the African Region (AFR) accounting for half of the studies (57/113), followed by the Western Pacific (WPR) and South-East Asia regions (SEAR) with 21.24% (24/113) and 15.93% (18/113), respectively (Fig. 2b). The smallest and largest study samples had 47 and 17,765 participants, respectively, with an average sample size of 1726 (interquartile range [312–1724]). Studies from AFR (*n* = 17,765), the Eastern Mediterranean (EMR) (*n* = 16,075) and WPR (*n* = 13,980) reported the largest sample size (Additional file 3: Table S3). Forty-nine studies (43.36%) comprised both symptomatic and asymptomatic participants, whereas 26.55% and 30.09% included respectively asymptomatic and symptomatic participants only. Children under 15 years old were targeted in 10.62% of studies, mostly in AFR (*n* = 11); pregnant women were investigated in 7.08% in the African Region (*n* = 7) [18–24] and Region of the Americas (AMR) (*n* = 1) [25], and one study (0.88%) was conducted among 4570 women of childbearing age in the Democratic Republic of the Congo (DRC) [26] (Additional file 3: Table S3).

All included studies used molecular diagnostic methods including nested polymerase chain reaction (PCR) (71.43%), real-time polymerase chain reaction (RT-PCR) (10.71%), quantitative polymerase chain reaction (qPCR) (4.46%) and loop-mediated isothermal amplification (LAMP) (3.54%) for *Plasmodium* spp. identification. Nested PCR with four pairs of species-specific primers (*P. falciparum*, *P. vivax*, *P. malariae* and *P. ovale* spp.) were seen in the majority of studies (*n* = 108), and a few also used *P. knowlesi*-specific primers for species identification and confirmation [27–31]. Only 7.07% of the studies used sympatric species-specific primers of *P*. *ovale curtisi* and *P. ovale wallikeri* [9, 10, 21, 32–36]*.* The target genes for the PCR assay were mainly 18S rRNA (83.19%), cytochrome b (9.73%) and plasmepsin 4 (1.17%) genes [33, 37]. One study from South-East Asia targeted 18S rRNA and cytochrome b genes simultaneously [38] (Additional file 3: Table S3).

### *P. malariae* and *P. ovale* spp. prevalence

The aggregate prevalence of *P. malariae* in the 102 studies using the random-effects model was 2.01% (95% CI 1.31–2.85%, tau^2^ = 0.07), with high and significant heterogeneity between the studies (*I*^2^ = 99.19%, *p* < 0.001). In comparison to *P. malariae*, the prevalence of *P. ovale* spp. using the same model was found to be 0.77% (95% CI 0.50–1.10%, tau^2^ = 0.02), with significant heterogeneity (*I*^2^ = 97.85%, *p* < 0.001) (Additional file 3: Tables S4 and S5).

### Subgroup analysis

#### Influence of WHO region

The AFR revealed the highest prevalence of the two species studied, 3.16% (95% CI 2.00–4.56%) for *P. malariae* and 1.69% (95% CI 1.11–2.38%) for *P. ovale* spp. The EMR showed the lowest prevalence of *P. malariae* of 0.06% (95% CI 0–0.52%) and no cases of *P*. *ovale* spp. The AMR had the second highest *P. malariae* prevalence of 2.94% (95% CI 0–12.56%). The differences among regions were found to be statistically significant (*I*^2^ = 97.85%, *p* < 0.001) (Table 1).

**Table 1:** Distribution of *P. malariae* and *P. ovale* spp. by WHO region

WHO region	*P. malariae*			*P. ovale* spp.		
	No. of studies	Prevalence % [95% CI]	*p* value	No. of studies	Prevalence % [95% CI]	*p* value
Africa	50	3.16 [2–4.56]	< 0.001	50	1.69 [1.11–2.38]	< 0.001
Western Pacific	21	0.88 [0.24–1.87]		20	0.33 [0.06–0.75]	
South-East Asia	17	1.04 [0.33–2.08]		16	0.05 [0.00–0.17]	
Eastern Mediterranean	5	0.06 [0–0.52]		5	No case	
Americas	9	2.94 [0–12.56]		4	0.00 [0–0.01]	

#### Influence of participants’ status: symptomatic *versus* asymptomatic

**Fig. 3:**
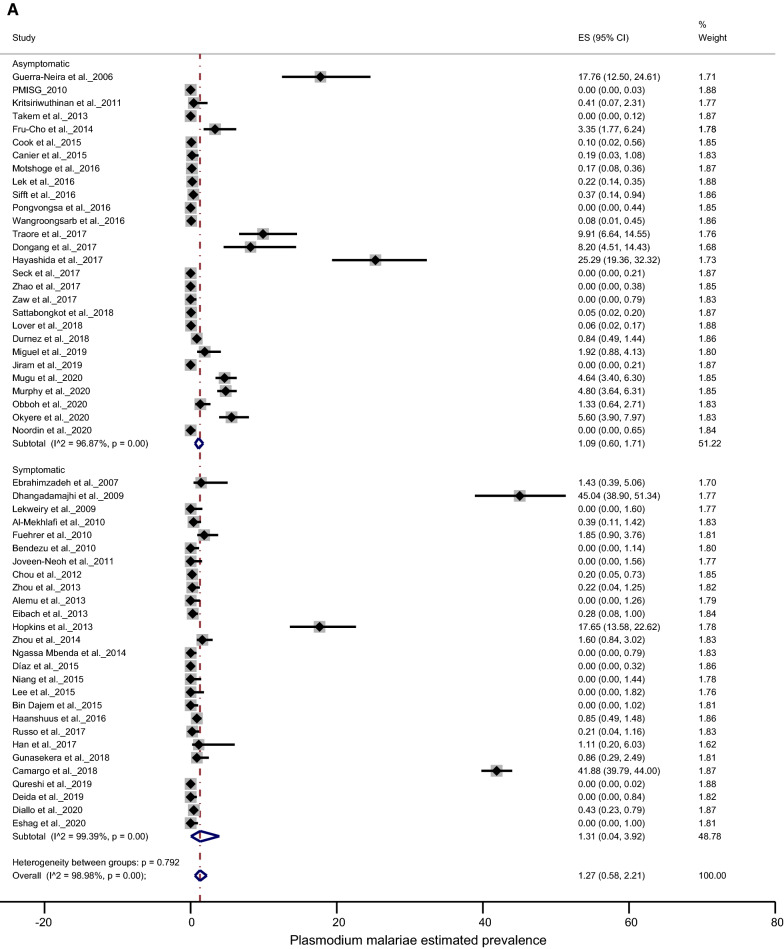

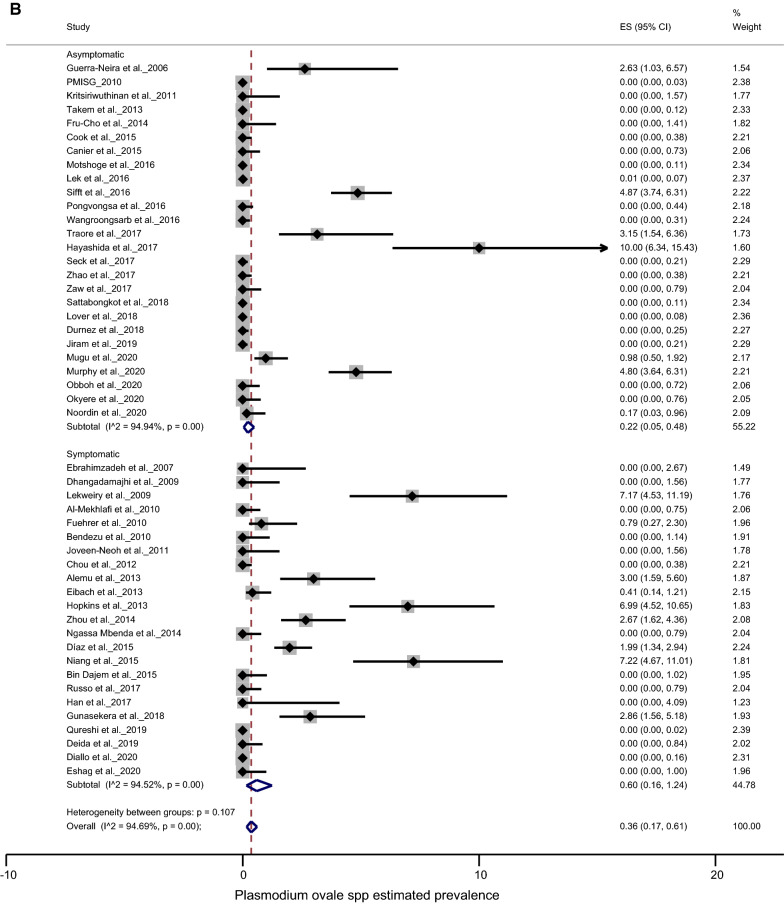
Forest plots of prevalence between asymptomatic and symptomatic participants: **a** *P. malariae*; **b** *P. ovale* spp. The prevalence of each study is represented by a spot and the horizontal bar centred by the prevalence gives the dispersion of the data

The prevalence of *P. malariae* was found to be lower in asymptomatic than in symptomatic subjects, 1.31% (95% CI 0.60–1.71%) and 1.09% (95% CI 0.04–3.92%), respectively, with no significant difference (*p* = 0.792) (Fig. 3a). Similarly, there was no significant difference (*p* = 0.107) for *P. ovale* spp. infection between symptomatic 0.60% (95% CI 0.16–1.24%) and asymptomatic subjects 0.22% (95% CI 0.05–0.48%) (Fig. 3b).

#### Prevalence among children, adults and pregnant women

**Fig. 4:**
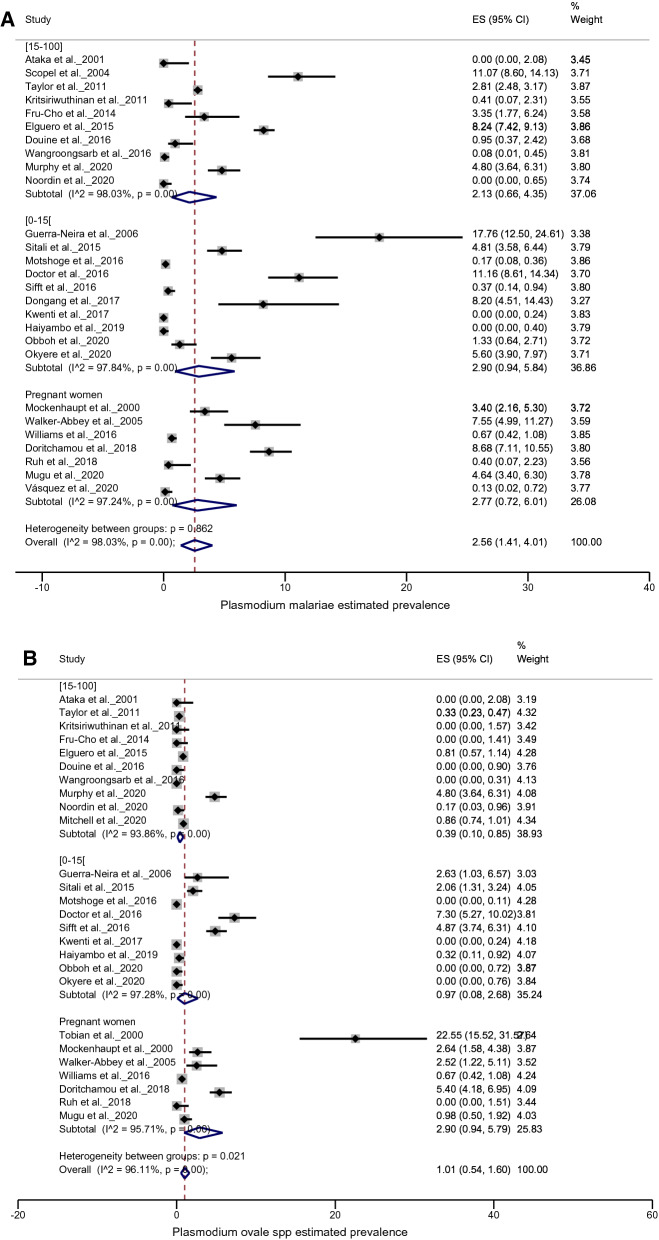
Forest plots of *Plasmodium* prevalence in studies conducted in specific groups (pregnant women, children under 15 and participants over 15 years old). **a** *P. malariae* prevalence, **b** *P. ovale* spp. prevalence. The prevalence of each study is represented by a spot and the horizontal bar centred by the prevalence gives the dispersion of the data

On analysis, the *P. malariae* species was found to be equally distributed among adults (2.13% with 95% CI 0.66–4.35%), children (2.90% with 95% CI 0.94–5.84%) and pregnant women (2.77% with 95% CI 0.72–6.01%) (*p* = 0.862). In contrast, the *P. ovale* spp. were seen to be more prevalent in pregnant women 2.90% (95% CI 0.94–5.79%) than in children ≤ 15 years 0.97% (95% CI 0.08–2.66%) and in patients > 15 years old 0.39% (95% CI 0.10–0.85%) (*p* = 0.021) (Figs. 4a, b).

### *P. ovale wallikeri* and *P. ovale curtisi *prevalence

For *P. ovale* spp. pooled prevalence, only five out of 95 included studies clearly revealed *P. ovale* subspecies prevalence. In these studies, *P. ovale curtisi* (1.65% with 95% CI 0.14–4.73%) appeared to be more prevalent than *P. ovale wallikeri* (1.55% with 95% CI 0.38–3.47%). However, there was no significant difference between them (*p* = 0.698) (Additional file 4).

### Mixed infection prevalence

The prevalence of *P. malariae* co-infections with at least one of the human *Plasmodium* species was 2.14% (95% CI 1.32–3.15%) versus 0.85% (95% CI 0.59–1.16%) in mono-infections, whereas *P. ovale* spp. showed a prevalence of 1.84% (95% CI 1.16–2.69%) in co-infections and 0.66% (95% CI 0.43–0.94%) in mono-infections. Analysis of the RD showed that both species were more likely to be present in co-infections than mono-infections in the studied subjects with RD value 4.16% (95% CI 1.59–6.73%, *p* = 0.002) for *P. malariae* and 5.05% (95% CI 2.40–7.69%, *p* < 0.001) for *P*. *ovale* spp. (Additional file 5).

### Meta-regression and publication bias

#### Evolution of prevalence over the years

**Fig. 5:**
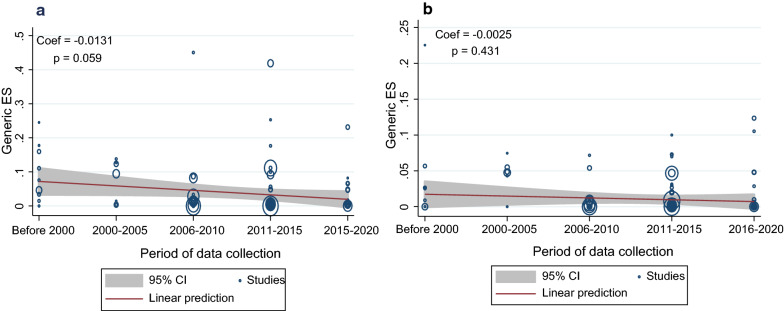
Meta-regression of prevalence over the period of data collection: **a** meta-regression of *P. malariae* prevalence, **b** meta-regression of *P. ovale* spp. prevalence

The evolution of *P. malariae* and *P. ovale* spp. prevalence over the years was explored by meta-regression. Globally, a negative correlation between pooled prevalence and years of data collection was observed, though it was not significant for either *P. malariae* (Coef = −0.0131; *p* = 0.059) or *P. ovale* spp. (Coef = −0.0025; *p* = 0.431) (Fig. 5a, b). This observation was also the same for each region separately except for the WPR, where the *P. malariae* prevalence had declined significantly (Coef = −0.0212, *p* < 0.001) (Additional file 6).

#### Exploration of other potential confounders

According to the results, none of the variables of sample size, year of publication or period of data collection was a significant moderator in estimation of either *P. malariae* or *P. ovale* spp. prevalence; therefore, no evidence of publication bias was observed in the estimated prevalence due to these factors (Table 2). In addition, the funnel plot does not indicate any evidence of publication bias for either of the two species. However, the exact Egger’s test for small-study effect was significant for *P. ovale* spp. (*p* = 0.046), where the small study size may be the reason for bias towards *P. ovale* spp. prevalence (Additional file 7).

**Table 2:** Meta-regression of potential confounders: sample size, date of publication, period of data collection

Species	Cofactors	Coef	[95% confidence interval]		*z*	*p* value
*P. malariae*	Sample size	− 2.36E−06	− 7.24E−06	2.51E−06	− 0.95	0.342
	Date of publication	− 0.0021299	− 0.0051524	0.0008927	− 1.38	0.167
	Period of data collection	− 0.0131058	− 0.0266983	0.0004866	− 1.89	0.059
*P. ovale* spp.	Sample size	− 4.95E−07	− 1.44E−06	4.45E−07	− 1.03	0.302
	Date of publication	− 0.0003042	− 0.0015801	0.0009716	− 0.47	0.640
	Period of data collection	− 0.0025502	− 0.008897	0.0037966	− 0.79	0.431

## Discussion

In the past two decades, WHO has reported a slight decline in malaria cases, from 238 million cases in 2000 to 229 million in 2019, with incidence declining from 363 to 225 cases per 1000 population through increased coverage of preventive and control measures (particularly long-lasting insecticide-treated nets, early diagnosis using rapid diagnostic tests, global improvement of WHO's policy development and dissemination processes for malaria control) [1, 39, 40]. These have reflected in the regression of malaria and even its eradication in some countries during this period, notably in the United Arab Emirates, Morocco, Armenia, Argentina, Paraguay, Algeria, Uzbekistan, Sri Lanka, Turkmenistan and Kyrgyzstan [1]. This systematic review and meta-analysis estimated the disease trend of *P. malariae* and *P. ovale* spp*.* from January 2000 to December 2020.

The overall prevalence of *P. malariae* infections (2.01%) was higher as compared to *P. ovale* spp. (0.77%), with AFR being the most frequently affected for both species. Epidemiological studies on malaria conducted in the AMR, SEAR and EMR did not very often pay attention to *P. malariae* and/or *P. ovale* spp. due to their low prevalence/severity [12, 41–46], yet some studies report *P. malariae* prevalence of more than 40% in these regions [12, 47]. This may be the reason for the underestimation of *P. malariae* and *P. ovale* spp. prevalence rates in these regions, as observed in this review. Negative correlations obtained in the meta-regression of prevalence as a function of data collection period indicate that the prevalence of both *P. malariae* and *P. ovale* spp. species have declined over the last 20 years, but not significantly. WHO has several strategic programmes to eliminate malaria, with a particular focus on *P. falciparum* and *P. vivax* malaria but none specific to *P. malariae* or *P. ovale* spp., which may have resulted in a low regression observed for these two species with a significant decline of *P. falciparum* and *P. vivax* prevalence recorded globally [39, 48].

Gneme et al*.* [49], in a 4-year longitudinal study conducted in Burkina Faso from 2007 to 2010, revealed that the prevalence of *P. malariae* increased 15-fold and that of *P. ovale* spp. increased fourfold. Moreover, in the last 5 years, fairly high prevalence has been reported for *P. ovale* spp. [10, 13] and *P. malariae* [9, 10, 12] (> 10% and 20%, respectively) in AFR and AMR due to the increased usage of diagnostic PCR assay [50]. The *P. ovale* spp. was reported in 12 out of 26 provinces of the DRC during 2007. Six years later, in 2013, the demographic and health survey revealed *P. ovale* spp. in 24/26 provinces of DRC, with an increased prevalence in several provinces [51]. This reflects a spread of *P. ovale* spp. that urgently needs to be addressed, as DRC is the second most affected country by malaria in the world [1]. Several studies have also reported an equal distribution of *P. ovale* subspecies in AFR [21, 32, 35, 51]. High prevalence of asymptomatic malaria including *P. malariae* [9, 34, 42, 52–54] and *P. ovale* spp. [9, 35, 54, 55] infections were reported in many studies. Our analysis indicates that both species showed the same distribution between symptomatic and asymptomatic subjects [56]. *P. malaria* and *P. ovale* spp. are often misidentified as *P. falciparum* and *P. vivax,* respectively, which complicates the routine diagnosis [8, 57] and further leads to asymptomatic disease reservoirs [36] which could jeopardize malaria elimination programmes and therefore merits more attention.

Children and pregnant women are well known as vulnerable groups to malaria infection. Woldearegai et al. [10] reported in a Gabonese population that immunity to *P. malariae* builds up with age similarly to *P. falciparum*, but incompletely due to lower parasitaemia, which induces limited immune stimulation. In this study, subgroup analysis showed that *P. malariae* appears to have the same distribution in children, adults and pregnant women, whereas the highest *P. ovale* spp. prevalence among the subgroups was observed in pregnant women. Knowing that *P. malariae* has the same therapeutic protocol as *P. falciparum* [3, 58], the use of intermittent preventive treatment in pregnancy (ITPp) contributes to the reduction of *P. malariae* burden in this group, unlike *P. ovale* spp., which can remain dormant in the liver (hypnozoites stage) for long periods. A cross-sectional study including 4570 women of childbearing age conducted by Taylor et al*.* [26] found that *P. malariae* was more prevalent in non-pregnant than pregnant women in the Wocba region (DRC). Unfortunately, this observation could not be linked with any factor, as the authors had no data on ITPp or antenatal care. On the other hand, Mitchell and colleagues reported *P. ovale* spp. prevalence twice as high in men (mainly miners) as in women, suggesting that male susceptibility was possibly due to the high exposure in their work environment. Therefore, the conclusions as to the risk groups for these two species remain controversial.

In the reported meta-analysis, the difference in risk of co-infections with another *Plasmodium* species reveals that both *P. malariae* and *P. ovale* spp. occurred more in co-infections, as reported earlier [9, 10, 13, 59–62]. The recurrence of the coexistence of two or more *Plasmodium* species can be explained by the density-dependent regulation model proposed by Bruce and Day [63, 64]. This model suggests that when an individual carrying a *Plasmodium* species is reinfected by a second species, the parasitaemia due to the first species is down-regulated. However, when the species with the highest parasitaemia exceeds a certain threshold, the host immune response is triggered to control the infection. Once the infection is under control, the system is deactivated, promoting the growth of the second infecting species. This cycle is believed to be responsible for the persistence of several species in the host. In addition, it is also known that *P. malariae* and *P. ovale* spp. preferentially develop in mature and younger red blood cells respectively [65, 66]. This differential target of invasion and no antagonism mechanism reported between these two species could explain their co-existence in an individual. Obviously, this requires further research, as not much data is available in this regard. Although not considered a significant threat to malaria elimination, the insufficient knowledge of the biological dynamics of *P. malariae* and *P. ovale* spp. should not be neglected because of their low prevalence and disease severity.

The strength of this study lies in the estimation of the prevalence rates of these two non-*falciparum* species based essentially on molecular techniques known to be the most sensitive diagnostic tool, and the global coverage of the WHO health regions in the last two decades, thus offering a global overview of *P. malariae* and *P. ovale* spp. malaria burden. However, the lack of whole information in some studies made it impossible to thoroughly analyse the data for identifying the environmental and biological factors that regulate the dynamics of these two species and which will be the subject of our future investigations.

### Limitations

However, this study had certain limitations. First, the pooled prevalence data was estimated from studies conducted in different geographic areas and at different times, as reflected in the high heterogeneity observed. Second, the AFR had ten times as many papers as some of the regions included, which could be a source of imbalance in pooled prevalence data. Third, not all studies mentioned the characteristics of the study areas to permit us to explore other sources of heterogeneity. Fourth, only papers published in English or French were included in this review. Finally, field or laboratory working conditions that were not always clearly detailed and/or sometimes differed could have generated several other confounding variables that affected our results.

## Conclusion

This study summarizes the distribution and the global trend of *P. malariae* and *P. ovale* spp. prevalence in the last two decades. Based on our results, the prevalence of both species has declined slightly over this period, although high prevalence has been recorded in AFR and the AMR recently. Both species are equally prevalent in asymptomatic and symptomatic individuals of all age groups, which supports the interest of their inclusion in the new strategies for prevention, treatment and malaria elimination programmes.

## Supplementary Information

**Additional file 1. **PRISMA Checklist.

**Additional file 2. **Search strategy.

**Additional file 3. Table S1.** Score of included study. **Table S2.** Studies excluded from meta-analysis. **Table S3.** Characteristics of included studies. **Table S4.** *Plasmodium malariae* pooled prevalence. **Table S5.*** Plasmodium ovale *in future. pooled prevalence.

**Additional file 4. **Comparison of *P. ovale curtisi* and *P. ovale wallikeri* prevalence.

**Additional file 5. **Risk difference (RD) mono-infection vs mix infection.

**Additional file 6. **Meta-regression of prevalence over the period of data collection by region.

**Additional file 7. **Funnel plot for publication bias and Egger’s test for small study effects.

## Data Availability

All data generated or analyzed during this study are included in this published article and its additional files.

## Abbreviations

AFR: African Region
AMR: Region of the Americas
DRC: Democratic Republic of the Congo
EMR: Eastern Mediterranean Region
JBI: Joanna Briggs Institute
LAMP: Loop-mediated isothermal amplification
PCR: Polymerase chain reaction
PRISMA: Preferred Reporting Items for Systematic Reviews and Meta-Analyses
qPCR: Quantitative polymerase chain reaction
RD: Risk difference
RT-PCR: Real-time polymerase chain reaction
SEAR: South-East Asia Region
WPR: Western Pacific Region
