# Supplementary material for: Global trend of Plasmodium malariae and Plasmodium ovale spp. malaria infections in the last two decades (2000–2020): a systematic review and meta-analysis
Source: Parasit Vectors. 2021 Jun 3;14:297. doi: 10.1186/s13071-021-04797-0 (PMC8173816; doi:10.1186/s13071-021-04797-0)
Supplement: Supplementary file 2 — Additional file 2. Search strategy. [file 13071_2021_4797_MOESM2_ESM.docx]

**Supplementary file 2:** search strategy

**PubMed search strategy**

((((((((((((((("malaria"[MeSH Terms] OR "malaria"[All Fields]) OR "malarias"[All Fields]) OR "malaria s"[All Fields]) OR "malariae"[All Fields]) OR ((("plasmodium"[MeSH Terms] OR "plasmodium"[All Fields]) OR "plasmodiums"[All Fields]) OR "plasmodium s"[All Fields])) OR "plasmodium malariae"[MeSH Terms]) OR "plasmodium ovale"[MeSH Terms]) OR "plasmodium ovale"[MeSH Terms]) OR (((("malaria"[MeSH Terms] OR "malaria"[All Fields]) OR "malarias"[All Fields]) OR "malaria s"[All Fields]) OR "malariae"[All Fields])) OR ("ovale"[All Fields] OR "ovales"[All Fields])) OR ("ovale"[All Fields] OR "ovales"[All Fields])) OR "wallikeri"[All Fields]) OR "curtisi"[All Fields]) OR ("non"[All Fields] AND ((("malaria, falciparum"[MeSH Terms] OR ("malaria"[All Fields] AND "falciparum"[All Fields])) OR "falciparum malaria"[All Fields]) OR ("falciparum"[All Fields] AND "malaria"[All Fields])))) AND ((((("epidemiology"[MeSH Terms] OR (((("epidemiologies"[All Fields] OR "epidemiology"[MeSH Subheading]) OR "epidemiology"[All Fields]) OR "epidemiology"[MeSH Terms]) OR "epidemiology s"[All Fields])) OR ("proportion"[All Fields] OR "proportions"[All Fields])) OR (((((("epidemiology"[MeSH Subheading] OR "epidemiology"[All Fields]) OR "frequency"[All Fields]) OR "epidemiology"[MeSH Terms]) OR "frequence"[All Fields]) OR "frequences"[All Fields]) OR "frequencies"[All Fields])) OR "prevalence"[MeSH Terms]) OR ((((((((("epidemiology"[MeSH Subheading] OR "epidemiology"[All Fields]) OR "prevalence"[All Fields]) OR "prevalence"[MeSH Terms]) OR "prevalance"[All Fields]) OR "prevalences"[All Fields]) OR "prevalence s"[All Fields]) OR "prevalent"[All Fields]) OR "prevalently"[All Fields]) OR "prevalents"[All Fields]))) AND "PCR"[All Fields]

**Other data base search strategy**

"malaria" OR "plasmodium" OR "plasmodium malariae" OR "plasmodium ovale" OR "plasmodium ovales" OR "malariae" OR "ovale" OR "ovales" OR "wallikeri" OR "curtisi" OR "non falciparum malaria" AND "epidemiology" OR "epidemiology" OR "proportion" OR "frequence" OR "prevalence" AND "PCR"
