# Supplementary material for: Global trend of Plasmodium malariae and Plasmodium ovale spp. malaria infections in the last two decades (2000–2020): a systematic review and meta-analysis
Source: Parasit Vectors. 2021 Jun 3;14:297. doi: 10.1186/s13071-021-04797-0 (PMC8173816; doi:10.1186/s13071-021-04797-0)

Supplementary file 4: Comparison of *P. ovale curtisi* and *P. ovale wallikeri* prevalence

**Studies**	Estimate (95% C.I.)	Poc/N	Pow/N
Fançony et al. 2012	−0.0026 (−0.0266, 0.0215)	11/3316	12/3316
Williams et al. 2016	−0.0103 (−0.0379, 0.0173)	7/2526	10/2526
Hayashida et al. 2017	−0.0803 (−0.1866, 0.0260)	2/170	6/170
Woldearegai et al. 2019	0.0874 (0.0394, 0.1353)	74/834	38/834
Murphy et al. 2020	0.0085 (−0.0353, 0.0523)	15/1000	13/1000
**Overall (I^2=81.7 % , P=0.0040)**	**0.0082 (−0.0333, 0.0498)**	**109/7846**	**79/7846**

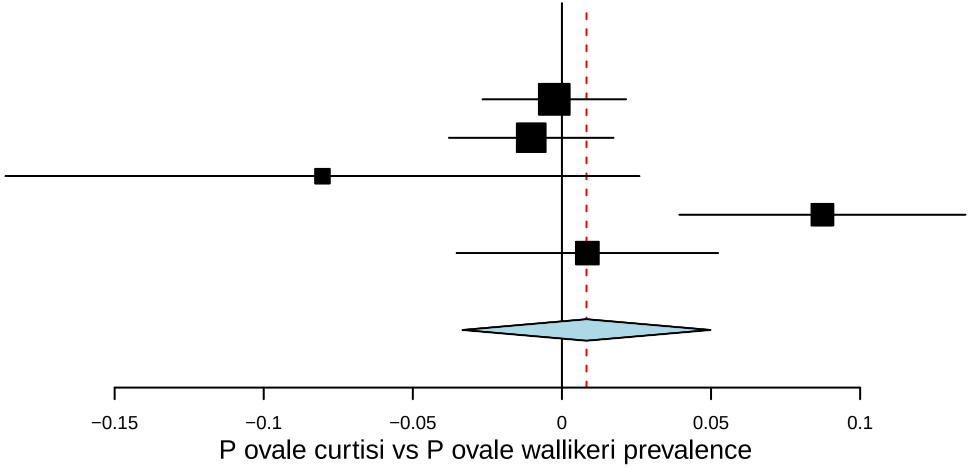

Supplement: Supplementary file 4 — Additional file 4. Comparison of P. ovale curtisi and P. ovale wallikeri prevalence. [file 13071_2021_4797_MOESM4_ESM.pdf]
