# Supplementary material for: Global trend of Plasmodium malariae and Plasmodium ovale spp. malaria infections in the last two decades (2000–2020): a systematic review and meta-analysis
Source: Parasit Vectors. 2021 Jun 3;14:297. doi: 10.1186/s13071-021-04797-0 (PMC8173816; doi:10.1186/s13071-021-04797-0)
Supplement: Supplementary file 5 — Additional file 5. Risk difference (RD) mono-infection vs mix infection. [file 13071_2021_4797_MOESM5_ESM.pdf]

Supplementary file 5: Risk difference (RD) mono infection vs mix infection. Both species, *P. malariae* and *P. ovale spp* seem to be more prevalent in mix infection compare to mono infection. a)*P. malariae* b) *P. ovale spp*

**Studies**	Estimate (95% C.I.)	MonoI/N	MixI/N
Tobian et al. 2000	−0.3106 (−0.4479, −0.1734)	3/102	22/102
Mehlotra et al. 2000	0.0796 (0.0498, 0.1094)	75/2162	25/2162
Mockenhaupt et al. 2000	−0.1571 (−0.2172, −0.0969)	0/530	18/530
Toma et al. 2001	−0.0174 (−0.0930, 0.0582)	2/336	3/336
R K. Mehlotra et al. 2002	−0.1446 (−0.1982, −0.0911)	28/670	79/670
Scopel et al. 2004	−0.0849 (−0.1470, −0.0227)	18/497	37/497
Walker−Abbey et al. 2005	−0.2389 (−0.3219, −0.1560)	0/278	21/278
Guerra−Neira et al. 2006	−0.0816 (−0.1940, 0.0308)	10/152	17/152
Kasehagen et al. 2006	−0.0966 (−0.1369, −0.0562)	46/1182	100/1182
Curado et al. 2006	0.0290 (−0.0486, 0.1066)	1/318	0/318
Ebrahimzadeh et al. 2007	0.0740 (−0.0427, 0.1907)	2/140	0/140
Bruce et al. 2008	−0.2152 (−0.2408, −0.1895)	21/2918	255/2918
Dhangadamajhi, Kar, et Ranjit 2009	−0.1446 (−0.2337, −0.0555)	40/242	69/242
Al−Mekhlafi et al. 2010	−0.0387 (−0.0999, 0.0226)	0/511	2/511
Fuehrer et al. 2010	−0.0138 (−0.0850, 0.0574)	3/379	4/379
Steenkeste et al. 2010	−0.3074 (−0.4267, −0.1881)	0/134	17/134
Taylor et al. 2011	−0.0053 (−0.0258, 0.0153)	30/4570	34/4570
Taylor et al.(b) 2011	−0.0174 (−0.0321, −0.0026)	106/8838	142/8838
Kritsiriwuthinan et al. 2011	0.0333 (−0.0557, 0.1224)	1/241	0/241
Fançony et al. 2012	−0.0840 (−0.1081, −0.0600)	3/3316	43/3316
Chou et al. 2012	−0.0276 (−0.0714, 0.0162)	0/1000	2/1000
Zhou et al. 2013	0.0244 (−0.0409, 0.0898)	1/449	0/449
Kaisar et al. 2013	0.0579 (0.0223, 0.0936)	22/1509	6/1509
Eibach et al. 2013	0.0324 (−0.0189, 0.0838)	2/727	0/727
Hopkins et al. 2013	−0.3923 (−0.4762, −0.3084)	0/272	48/272
Fru−Cho et al. 2014	−0.0145 (−0.0990, 0.0700)	4/269	5/269
Sluydts et al. 2014	−0.0102 (−0.0284, 0.0080)	8/5793	13/5793
Zhou et al. 2014	−0.0304 (−0.0888, 0.0281)	3/562	6/562
Edwards et al. 2015	0.0073 (−0.0172, 0.0318)	2/3205	1/3205
Sitali et al. 2015	−0.1424 (−0.1893, −0.0955)	4/873	38/873
Cook et al. 2015	0.0124 (−0.0052, 0.0299)	21/6233	13/6233
Sitali et al. 2015	−0.1424 (−0.1893, −0.0955)	4/873	38/873
Cook et al. 2015	0.0164 (−0.0274, 0.0602)	1/1000	0/1000
Canier et al. 2015	−0.0227 (−0.0833, 0.0380)	0/521	1/521
Parker et al. 2015	0.0221 (−0.0371, 0.0813)	1/547	0/547
Elguero et al. 2015	0.1064 (0.0844, 0.1284)	90/3958	8/3958
Li et al. 2016	0.0272 (0.0006, 0.0539)	4/2701	0/2701
Motshoge et al. 2016	0.0306 (0.0076, 0.0536)	6/3624	0/3624
Lek et al. 2016	0.0396 (0.0238, 0.0554)	17/7707	0/7707
Doctor et al. 2016	−0.2194 (−0.2836, −0.1552)	5/466	47/466
Baum et al. 2016	−0.0103 (−0.0448, 0.0242)	1/1615	2/1615
Williams et al. 2016	−0.0400 (−0.0676, −0.0125)	3/2526	14/2526
Papa Mze et al. 2016	0.0356 (−0.0596, 0.1308)	1/211	0/211
Idris et al. 2016	0.1064 (0.0929, 0.1200)	133/10430	0/10430
Douine et al. 2016	0.0358 (−0.0318, 0.1033)	3/421	1/421
Wangroongsarb et al. 2016	0.0147 (−0.0246, 0.0540)	1/1242	0/1242
Haanshuus et al. 2016	0.0338 (−0.0031, 0.0707)	9/1412	3/1412
Traore et al. 2017	0.0629 (−0.0302, 0.1559)	14/222	8/222
Dongang et al. 2017	−0.0415 (−0.1670, 0.0839)	4/122	6/122
Russo et al. 2017	0.0235 (−0.0394, 0.0865)	1/484	0/484
Hayashida et al. 2017	−0.3455 (−0.4518, −0.2392)	4/170	39/170
Han et al. 2017	0.0546 (−0.0907, 0.1998)	1/90	0/90
Guerra et al. 2017	−0.2008 (−0.2918, −0.1098)	5/232	27/232
Ghinai et al. 2017	0.0128 (−0.0214, 0.0470)	1/1638	0/1638
Oboh et al. 2018	−0.0321 (−0.0985, 0.0343)	12/436	17/436
Gunasekera et al. 2018	0.0623 (−0.0117, 0.1362)	3/350	0/350
Sattabongkot et al. 2018	0.0145 (−0.0085, 0.0374)	2/3650	0/3650
Lover et al. 2018	0.0058 (−0.0136, 0.0252)	2/5085	1/5085
Camargo et al. 2018	0.0443 (0.0141, 0.0745)	7/2106	0/2106
Doritchamou et al. 2018	−0.1577 (−0.2007, −0.1146)	14/1037	76/1037
Durnez et al. 2018	−0.0050 (−0.0403, 0.0303)	6/1540	7/1540
Ruh et al. 2018	−0.0327 (−0.1202, 0.0547)	0/250	1/250
Amoah et al. 2019	−0.1221 (−0.1734, −0.0707)	9/729	39/729
Woldearegai et al. 2019	−0.3284 (−0.3764, −0.2804)	18/834	175/834
Yman et al. 2019	−0.1590 (−0.1847, −0.1333)	43/2897	223/2897
Sitali et al. (b) 2019	−0.0183 (−0.0978, 0.0612)	2/304	3/304
Diallo et al. 2020	−0.0190 (−0.0477, 0.0098)	3/2326	7/2326
Mugu et al. 2020	−0.0493 (−0.0977, −0.0009)	13/819	25/819
Murphy et al. 2020	−0.0739 (−0.1178, −0.0301)	13/1000	35/1000
Obboh et al. 2020	0.0886 (0.0283, 0.1489)	7/528	0/528
Okyere et al. 2020	0.2092 (0.1473, 0.2712)	28/500	0/500
Vasquez et al. 2020	0.0185 (−0.0309, 0.0678)	1/787	0/787
**Overall (I^2=97.28 % , P< 1e−04)**	**−0.0416 (−0.0673, −0.0159)**	**948/115798**	**1823/115798**

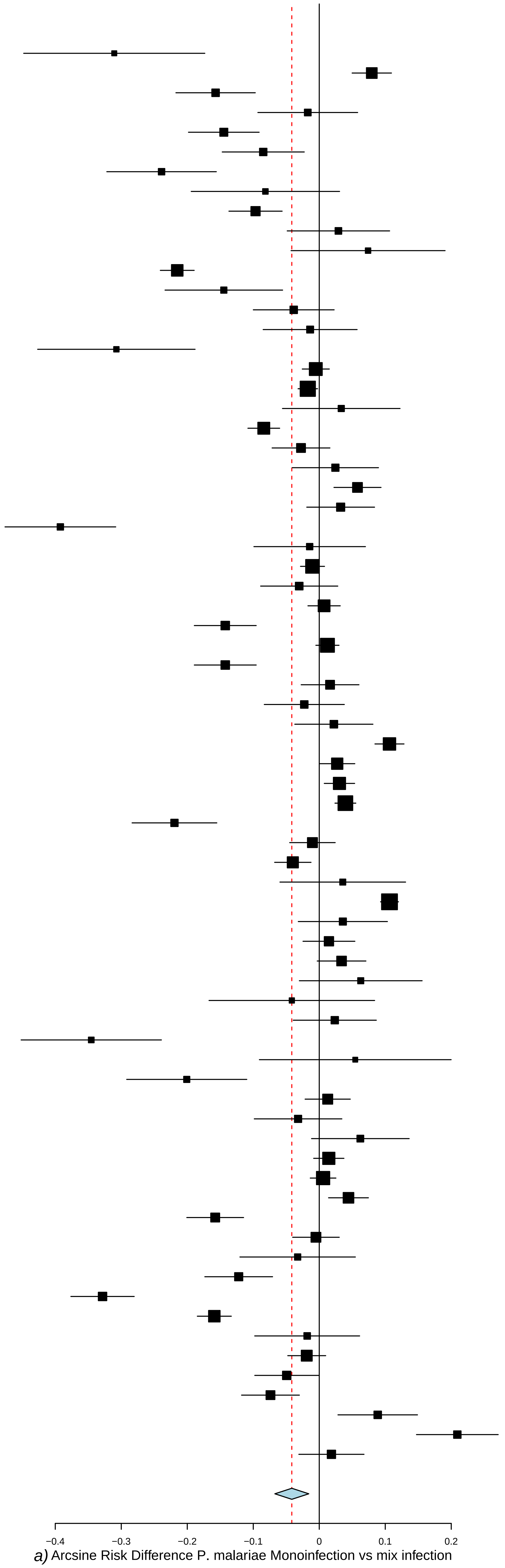

a) Arcsine Risk Difference P. malariae Monoinfection vs mix infection

Studies	Estimate (95% C.I.)	MonoI/N	MixI/N
Tobian et al. 2000	−0.1756 (−0.3128, −0.0383)	6/102	17/102
Mockenhaupt et al. 2000	−0.1138 (−0.1740, −0.0536)	1/530	13/530
Toma et al. 2001	0.0636 (−0.0119, 0.1391)	3/336	0/336
R K. Mehlotra et al. 2002	−0.1255 (−0.1791, −0.0720)	6/670	32/670
Walker−Abbey et al. 2005	−0.1224 (−0.2053, −0.0394)	0/278	7/278
Guerra−Neira et al. 2006	−0.0598 (−0.1722, 0.0527)	1/152	3/152
Kasehagen et al. 2006	−0.0943 (−0.1346, −0.0539)	15/1182	50/1182
Bruce et al. 2008	−0.1611 (−0.1868, −0.1355)	8/2918	131/2918
Lekweiry et al. 2009	0.1623 (0.0723, 0.2523)	15/237	2/237
Fuehrer et al. 2010	0.0598 (−0.0113, 0.1309)	3/379	0/379
Steenkeste et al. 2010	−0.1244 (−0.2441, −0.0046)	2/134	8/134
Taylor et al.(a) 2011	0.0192 (−0.0014, 0.0397)	28/4570	16/4570
Taylor et al.(b) 2011	−0.0157 (−0.0304, −0.0009)	9/8838	20/8838
Fançony et al. 2012	−0.0236 (−0.0476, 0.0005)	7/3316	16/3316
Alemu et al. 2013	0.0417 (−0.0383, 0.1217)	6/300	3/300
Eibach et al. 2013	0.0432 (−0.0082, 0.0945)	3/727	0/727
Hopkins et al. 2013	−0.1995 (−0.2835, −0.1155)	1/272	18/272
Sluydts et al. 2014	−0.0408 (−0.0590, −0.0225)	0/5793	14/5793
Zhou et al. 2014	−0.0393 (−0.0978, 0.0191)	5/562	10/562
Díaz et al. 2015	0.1096 (0.0697, 0.1495)	23/1209	1/1209
Niang et al. 2015	−0.2317 (−0.3170, −0.1464)	0/263	19/263
Sitali et al. 2015	−0.0879 (−0.1348, −0.0410)	2/873	16/873
Cook et al. 2015	0.0111 (−0.0065, 0.0286)	2/6233	0/6233
Sitali et al. 2015	−0.0879 (−0.1348, −0.0410)	2/873	16/873
Elguero et al. 2015	−0.0243 (−0.0464, −0.0023)	10/3958	22/3958
Li et al. 2016	0.0224 (−0.0043, 0.0491)	3/2701	0/2701
Lek et al. 2016	0.0059 (−0.0099, 0.0217)	1/7707	0/7707
Doctor et al. 2016	−0.1805 (−0.2447, −0.1163)	3/466	31/466
Baum et al. 2016	−0.0290 (−0.0634, 0.0055)	0/1615	3/1615
Williams et al. 2016	−0.0245 (−0.0520, 0.0031)	5/2526	12/2526
Idris et al. 2016	−0.1185 (−0.1320, −0.1049)	71/10430	416/10430
Traore et al. 2017	0.0181 (−0.0749, 0.1111)	4/222	3/222
Hayashida et al. 2017	−0.0682 (−0.1745, 0.0381)	6/170	11/170
Guerra et al. 2017	−0.1326 (−0.2235, −0.0416)	1/232	9/232
Oboh et al. 2018	−0.0558 (−0.1221, 0.0105)	0/436	3/436
Gunasekera et al. 2018	0.1361 (0.0621, 0.2101)	10/350	0/350
Doritchamou et al. 2018	−0.0359 (−0.0789, 0.0071)	22/1037	34/1037
Amoah et al. 2019	−0.0991 (−0.1504, −0.0478)	7/729	28/729
Woldearegai et al. 2019	−0.2462 (−0.2942, −0.1982)	8/834	95/834
Yman et al. 2019	−0.0958 (−0.1216, −0.0701)	28/2897	108/2897
Haiyambo et al. 2019	−0.0377 (−0.0826, 0.0072)	0/952	3/952
Sitali et al. (b) 2019	−0.1558 (−0.2353, −0.0763)	6/304	26/304
Mugu et al. 2020	−0.0177 (−0.0661, 0.0308)	3/819	5/819
Murphy et al. 2020	0.0262 (−0.0176, 0.0701)	28/1000	20/1000
**Overall (I^2=96.1 % , P< 1e−04)**	**−0.0505 (−0.0769, −0.0240)**	**364/80132**	**1241/80132**

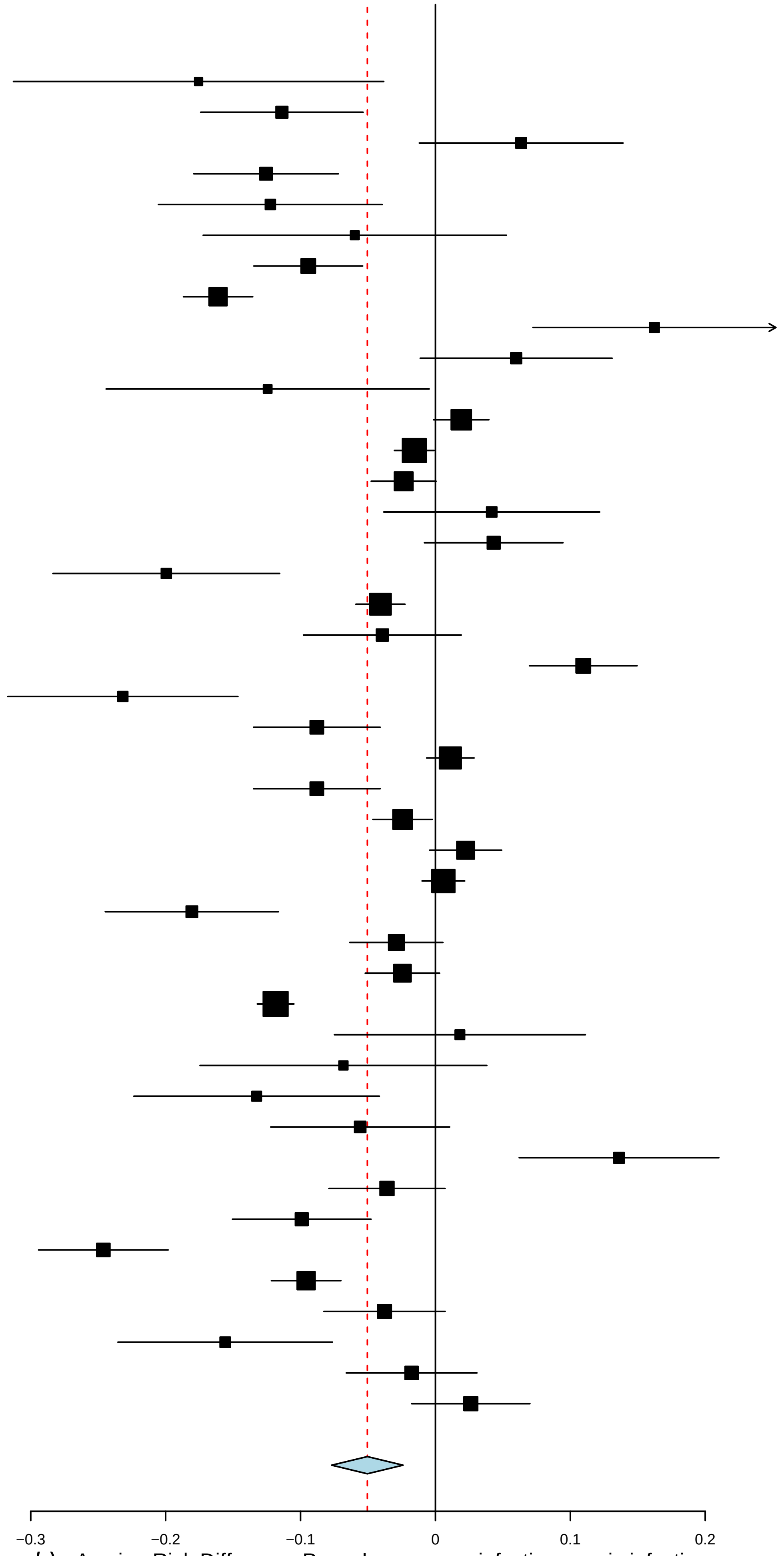

b) Arcsine Risk Difference P. ovale spp monoinfection vs mix infection
