# Supplementary material for: Global trend of Plasmodium malariae and Plasmodium ovale spp. malaria infections in the last two decades (2000–2020): a systematic review and meta-analysis
Source: Parasit Vectors. 2021 Jun 3;14:297. doi: 10.1186/s13071-021-04797-0 (PMC8173816; doi:10.1186/s13071-021-04797-0)
Supplement: Supplementary file 6 — Additional file 6. Meta-regression of prevalence over the period of data collection by region. [file 13071_2021_4797_MOESM6_ESM.docx]

**Supplemetary file 6:** Meta-regression of prevalence over the period of data collection for *P. malariae* and *P. ovale* spp respectively on the left (a) and right side (b). Negative but non-significant correlation between pooled prevalence and years of data collection for both species in all WHO region except for *P. malariae* in the Western pacific.

1. **b)**
