# Supplementary material for: Global trend of Plasmodium malariae and Plasmodium ovale spp. malaria infections in the last two decades (2000–2020): a systematic review and meta-analysis
Source: Parasit Vectors. 2021 Jun 3;14:297. doi: 10.1186/s13071-021-04797-0 (PMC8173816; doi:10.1186/s13071-021-04797-0)
Supplement: Supplementary file 7 — Additional file 7. Funnel plot for publication bias and Egger’s test for small study effects. [file 13071_2021_4797_MOESM7_ESM.docx]

**Supplementary file 7:** Funnel plot for publication bias and Eggers test for small study effects. A: for *P.* *malariae* pooled prevalence and B: for *P.* *ovale* *spp* pooled prevalence. No evidence of publication bias in the estimation of *P.* *malariae* pooled prevalence (p = 0.0758). On contrary the small study could create a publication bias in the estimation of *P.* *ovale spp* pooled prevalence (p = 0.0462).

| Egger test A | |
| --- | --- |
| beta 1 | 0.68 |
| SE of beta1 | 0.385 |
| z | 1.78 |
| Prob > z | 0.0758 |

| Egger test B | |
| --- | --- |
| beta 1 | 0.38 |
| SE of beta1 | 0.189 |
| z | 1.99 |
| Prob > z | 0.0462 |
